# In-Band Asymmetry Compensation for Accurate Time/Phase Transport over Optical Transport Network

**DOI:** 10.1155/2014/408613

**Published:** 2014-04-10

**Authors:** Sammy Siu, Wen-Hung Tseng, Hsiu-fang Hu, Shinn-Yan Lin, Chia-Shu Liao, Yi-Liang Lai

**Affiliations:** Telecommunication Laboratories, Chunghwa Telecom Co., Ltd., No. 99, Dianyan Road, Taoyuan County, Yangmei City 32601, Taiwan

## Abstract

The demands of precise time/phase synchronization have been increasing recently due to the next generation of telecommunication synchronization. This paper studies the issues that are relevant to distributing accurate time/phase over optical transport network (OTN). Each node and link can introduce asymmetry, which affects the adequate time/phase accuracy over the networks. In order to achieve better accuracy, protocol level full timing support is used (e.g., Telecom-Boundary clock). Due to chromatic dispersion, the use of different wavelengths consequently causes fiber link delay asymmetry. The analytical result indicates that it introduces significant time error (i.e., phase offset) within 0.3397 ns/km in *C*-band or 0.3943 ns/km in *L*-band depending on the wavelength spacing. With the proposed scheme in this paper, the fiber link delay asymmetry can be compensated relying on the estimated mean fiber link delay by the Telecom-Boundary clock, while the OTN control plane is responsible for processing the fiber link delay asymmetry to determine the asymmetry compensation in the timing chain.

## 1. Introduction

Precise synchronization of clocks has become an important technique not only for the scientific researches but also for the modern daily life. For many industrial infrastructures, the demands for precise time/phase synchronization have greatly increased recently, for example, communication networks, the smart grid of electric power distribution systems [1], and the practice of providing time stamps for financial networks [2]. Traditional communication network synchronization has relied on the accurate distribution of frequency [3]; evolving wireless networks require the distribution of accurate time/phase based on IEEE1588v2 for long term evolution (LTE) and accurate quality-of-service/service-level-agreement (QoS/SLA) measurements to determine the network health [4, 5].

**Figure 1:**
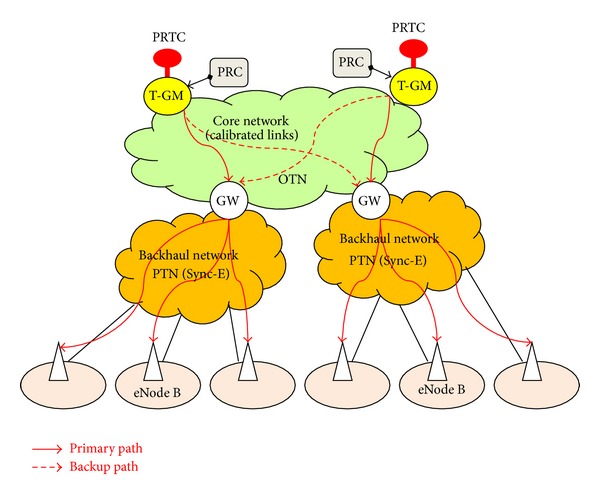
Architecture with centralized PRTC functions colocated with PRC. T-GM: telecom-grand master; GW: gate way.

The primary reference time clocks (PRTCs) location depends on the network that IEEE1588v2 support. Currently, the PRTCs are closer to the end application than than the primary reference clocks (PRCs) for traditional frequency distribution, in order to limit and control time/phase degradation [6]. The core networks will incorporate the accurate time/phase distribution capability into optical transport network (OTN), as addressed in ITU-T Recommendation G.709 [7]. The OTN provides new packet based time/phase distribution service; thus the PRTCs can colocate with the PRCs as shown in Figure 1. This architecture is compatible with PRTC redundancy (e.g., in order to secure the global navigation satellite system (GNSS) failures) and also requires a small number of GNSS receivers. The integrity of transferring accurate time/phase synchronization distribution over OTN in the core network and packet transport network with synchronous ethernet (PTN with Sync-E) in the backhaul networks [8, 9] can simplify network architecture, reduce operational expenditure (OPEX), and make the network easy to maintain.

**Figure 2:**
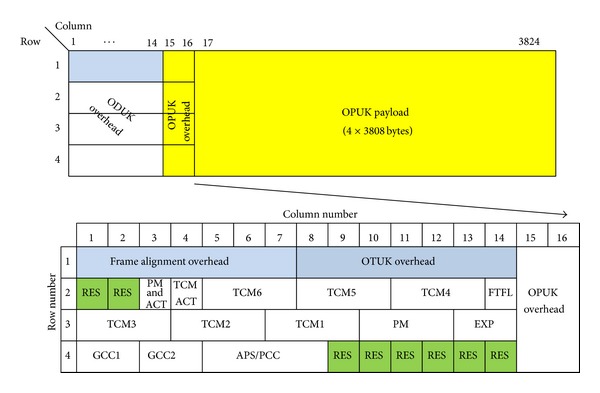
Optical transport network (OTN) frame format (ITU-T G.709).

For accurate time/phase transport over OTN, two options are considered: (1) the use of OTN optical channel data unit-k (ODUk) reserved overhead bytes to transport IEEE1588v2 Sync packets as shown in Figure 2 and (2) the use of optical supervisory channel (OSC) to transport IEEE1588v2 Sync packets [10]. The former belongs to In-band and the latter belongs to Out-of-band [7].

Nevertheless, each node and link can introduce asymmetry, which affects the adequate time/phase accuracy over the networks. Removal of packet delay variation (PDV) and asymmetry in the OTN nodes by means of IEEE1588v2 support (e.g., T-BC in every node [11, 12]) is analogue to backhaul network (ITU-T G.8275.1). In OTN, the forward and backward paths may not be the same wavelength depending on network configuration or wavelength switching; this will result in fiber link delay asymmetry and should be taken into account.

In this paper, we focus on the use of OTN overhead to transport Sync packets (In-band). The link delay asymmetry formation is given in Section 2, removal link and node asymmetry based on T-BC mode is given in Section 3, and the link delay asymmetry analysis is given in Section 4.

## 2. Use of Different Wavelengths

**Figure 3:**
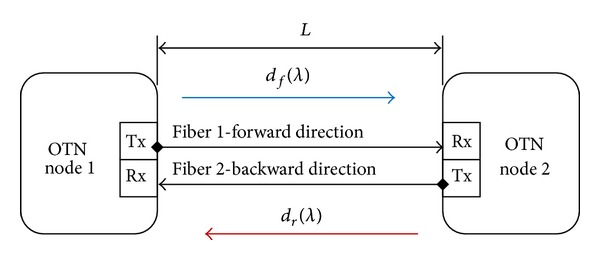
Packet signal transmission over optical fiber links.

In wavelength division multiplexing (WDM), multiple channels of information carried over the same fiber each using an individual wavelength to increase the transmission capacity as shown in Figure 3. Due to chromatic dispersion, the use of different wavelengths consequently causes fiber link delay asymmetry [13]. Group velocity is given by *v* = *c*/*η*, where *c* is speed of light and *η* is group refractive index depending on wavelength (*λ*). The fiber link delay asymmetry is given by

(1) A(λ)=df(λ)−dr(λ)=L×(ηf(λ)c−ηr(λ)c),

where *L* denotes the transmission distance (fiber link length), *d*_*f*_, *d*_*r*_ are forward and backward propagation delays, and *η*_*f*_, *η*_*r*_ are the related refractive indexes. The mean fiber link delay (*D*) can be represented as

(2) D=L2×(ηf(λ)c+ηr(λ)c).

Then,

(3) L=2Dηf(λ)/c+ηr(λ)/c.

Substituting *L* in (3) into (1) and simplifying, one obtains the fiber link delay asymmetry in terms of network mean fiber link delay as follows:

(4) A(λ)=df(λ)−dr(λ)=2D×(ηf(λ)−ηr(λ)ηf(λ)+ηr(λ)).

Half of the delay asymmetry (i.e.,  *A*(*λ*)/2) will contribute to the time error, where  *A*(*λ*) depends on the wavelength spacing.

## 3. Scheme to Remove Asymmetry Error of Node and Link

In IEEE1588v2 distribution, assume that the fiber link delay in each direction is symmetric, whereas in WDM systems the delay may not be symmetric. Fortunately, if a T-BC is implemented in every node in OTN, the mean fiber link delay *D* can be estimated by the T-BC mode, which would know the difference  (*d*_*f*_(*λ*) − *d*_*r*_(*λ*)) to compensate the phase offset  (*θ*) as shown in (4). The compensation scheme is proposed as follows.

### 3.1. Telecom-Boundary Clock Mode

**Figure 4:**
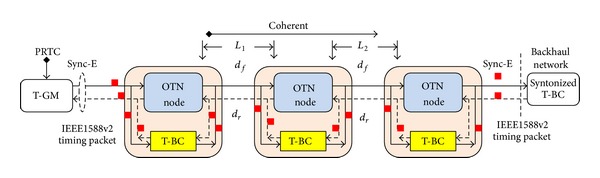
Telecom-Boundary clock mode in OTN.

**Figure 5:**
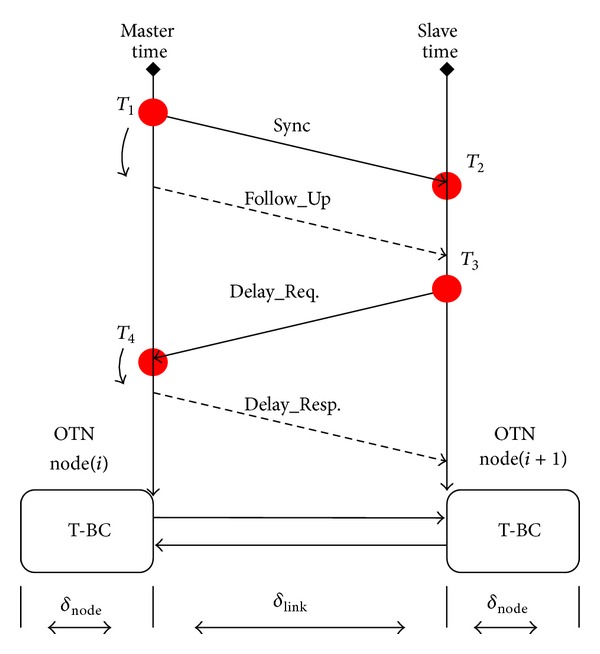
Time transfer mode based on T-BC mode.

Each node and link in a network can introduce asymmetry. In Telecom-Boundary clock (T-BC) mode [14], ingress/egress buffers are bypassed, and nodes asymmetry is avoided as shown in Figure 4. The time transfer model as shown in Figure 5 can be written as

(5) T2=T1+θ+δlink,M→S,T4=T3−θ+δlink,S→M,

where  *δ*_node_,  *δ*_link_  denote node and link delay, respectively, and assume  *δ*_node,*M*→*S*_ = *δ*_node,*S*→*M*_.

Based on the time transfer model in (5), the estimated mean fiber link delay $\hat{D}$ and estimated phase offset $\hat{\theta }$ can be derived as

(6) D^=δlink,M→S+δlink,S→M2=(T2−T1)+(T4−T3)2,

(7) θ^=(T2−T1)−(T4−T3)2−(δlink,M→S−δlink,S→M)2.

Equation (7) shows that any asymmetry will contribute with half of that to the error in the phase offset calculation. The second term in (7) is the link asymmetry compensation. The link asymmetry consists of mainly fiber link length asymmetry and fiber link delay asymmetry for use of different wavelengths. Substituting $\hat{D}$ in (6) into (4), assume that the fiber link length is symmetric; one obtains the fiber link delay asymmetry as

(8) A(λ)=((T2−T1)+(T4−T3))×(ηf(λ)−ηr(λ)ηf(λ)+ηr(λ)).

Substituting (8) into (7), one obtains the estimated phase offset $\hat{\theta }$ as

(9) θ^=((T2−T1)−(T4−T3)2)−12((T2−T1)+(T4−T3)) ×(ηf(λ)−ηr(λ)ηf(λ)+ηr(λ)).

The second term in (9) represents the fiber link delay asymmetry compensation. If the same wavelength is used both on forward and backward paths (i.e., *η*_*f*_(*λ*) = *η*_*r*_(*λ*)), then (9) becomes

(10) θ^=((T2−T1)−(T4−T3)2).

If there is a fiber length difference *β* between forward and backward paths, this will cause  *β* · *η*(*λ*)/2*c* error in the estimation of phase offset $\hat{\theta }$. For example, when *η* is 1.4682 at *λ* = 1550 nm, the estimated phase offset will have about 2.449 ns of error per meter of length asymmetry, which is related to the group delay (about 4.897 ns per meter).

### 3.2. Reducing Link Length Asymmetry

**Figure 6:**
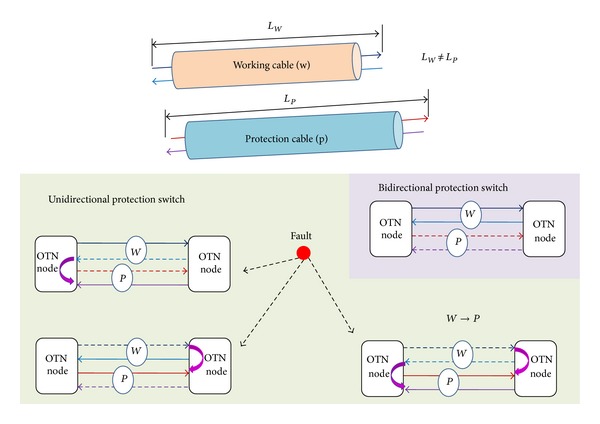
Schematic illustration of bidirectional/unidirectional network protection switches.

In a practical communication network, the link length asymmetry could be diminished to a tolerable extent if the fiber links are well designed at the beginning. An illustration of bidirectional and unidirectional protection switches in existent network fault management is shown in Figure 6. Bidirectional protection switch can minimize link length asymmetry (*β*) because two-way time transfer (TWTT) takes place within one cable. The cable asymmetry *β* should be within two meters (*β* < 2 m); this requires good cabling control. However, unidirectional protection switch TWTT takes place in separate cables, where the working and protection cables may not be in equal link length (i.e., *L*_*w*_ ≠ *L*_*p*_). In the current field trials, some budget is allocated for link length asymmetry unless the accurate link length asymmetry is manually measured and compensated.

## 4. In-Band Link Delay Asymmetry Analysis

The dispersion of single-mode optical fiber (e.g., SMF-28 that meets the requirements of ITU-T Recommendation G. 652) is

(11) D−(λ)=λSo8(1−λo4λ4),

where *S*_*o*_ (≤ 0.092 ps/(nm^2^·km)) is the zero dispersion slope, *λ*_*o*_ (1302 nm ≤*λ*_*o*_≤ 1322 nm) is the zero dispersion wavelength (*λ*_0_  = 1310 nm in the following calculation), and *λ*(1200 nm  ≤*λ* ≤ 1600 nm) is the operating wavelength [15]. The index of refraction *η* and $\overset{-}{D}(\lambda )$ are related by   $\left(1/c\right)\left(\partial \eta /\partial \lambda \right)=\overset{-}{D}(\lambda )$, which is then written as

(12) η(λ)c=η(λo)c+∫λoλD−(λ)dλ.

After integrating, we find that

(13) η(λ)c−η(λo)c=So8λ2(1−λo2λ2)2.

Substituting (13) into (1), the fiber link delay asymmetry per km  (*A*/*L*) is

**Figure 7:**
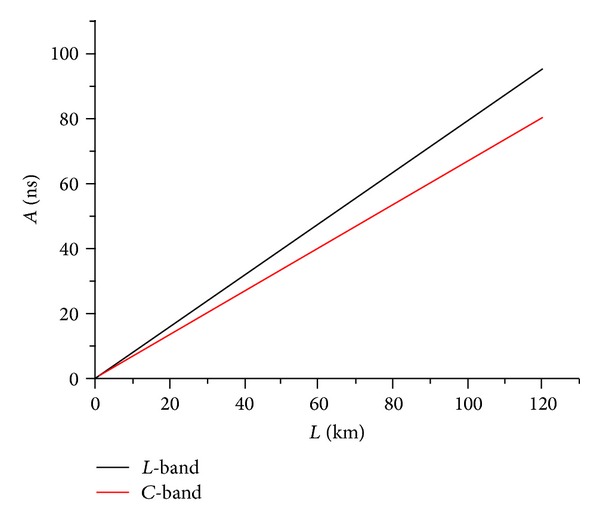
Fiber link delay asymmetry *A*(*λ*) versus transmission distance  *L*(km), where {*λ*_*f* = 1569 nm, *λ*_*r* = 1530 nm} in* C*-band and {*λ*_*f* = 1610 nm, *λ*_*r* = 1570 nm} in* L*-band.

(14) AL=ηf(λf)c−ηr(λr)c=So8{λf2(1−λo2λf2)2−λr2(1−λo2λr2)2},

where *λ*_*f*_ and *λ*_*r*_ are the wavelengths in the forward and backward directions and are defined based on ITU wavelength grid specification. The fiber link delay asymmetry *A*(*λ*) depends on the wavelength spacing {*λ*_*f*_, *λ*_*r*_} and also fiber link length (*L*) as shown in (14). The calculated values of *A*(*λ*)   versus  *L*(km) for {*λ*_*f*_ = 1569 nm, *λ*_*r*_ = 1530 nm} in* C*-band and {*λ*_*f*_ = 1610 nm, *λ*_*r*_ = 1570 nm} in* L*-band are depicted in Figure 7.

Based on (14), the maximum fiber link delay asymmetry for the two extreme wavelengths is about  *A*/*L* = 0.6795 ns/km in* C*-band (i.e., 1530 nm  ≤*λ* ≤ 1569 nm) and  *A*/*L* = 0.78854 ns/km in* L*-band (i.e., 1570 nm  ≤*λ* ≤ 1610 nm). This link delay asymmetry introduces significant time error (i.e., phase offset) within 0.3397 ns/km in* C*-band (e.g., *L* = 100 km, phase offset ≤33.97 ns) or 0.3943 ns/km in* L*-band (e.g., *L* = 100 km, phase offset  ≤39.43 ns).   The above results are summarized in Table 1.

For accurate time/phase transport, we have to take care of the fiber link delay asymmetry *A*(*λ*), especially for long haul transmission. Nevertheless, this error may be canceled out to some extent* relying* on the estimate $\hat{D}$ by T-BC (6). The OTN control plane contains global route information, which may play an important role in the asymmetry calibration process [10]. The network management system (NMS) is responsible for configuring the network including the wavelength (*λ*) assignment, collecting the mean fiber link delay $\left(\hat{D}\right)$ by the T-BC, and processing the fiber link delay asymmetry  (*A*(*λ*)) in (8) to determine the asymmetry compensation in the timing chain. The sum of  *A*(*λ*) in the timing chain can be written as

**Figure 8:**
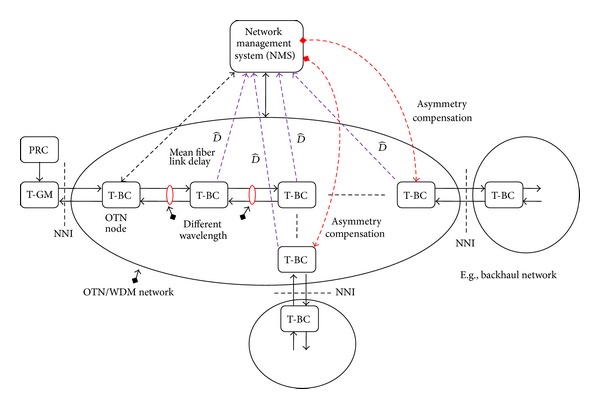
Asymmetry compensation support from control plane.

(15) AT(λf,λr)=A1(λf1,λr1)+A2(λf2,λr2) +⋯+AN(λfN,λrN),

where  *A*_*i*_(*λ*_*fi*_, *λ*_*ri*_) = 0 for *λ*_*fi*_ = *λ*_*ri*_. An illustration of asymmetry compensation support from OTN control plane is shown in Figure 8.

**Figure 9:**
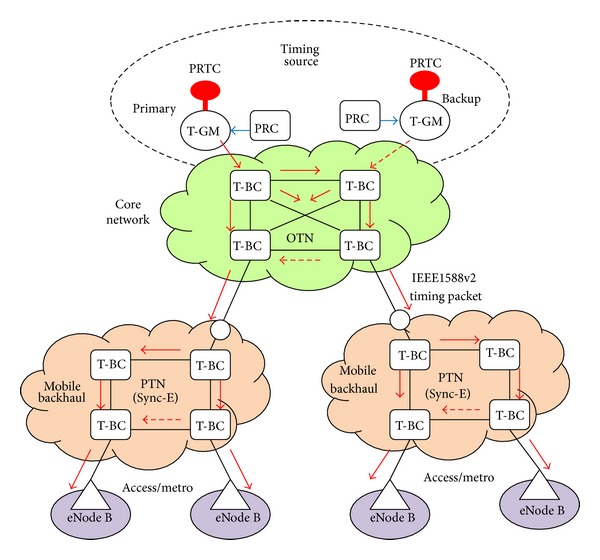
Time/phase over OTN networks with T-BC mode.

The integrity of transferring accurate time/phase synchronization over OTN and PTN with Sync-E networks is shown in Figure 9. Figure 9 is based on the full timing support (e.g., T-BC) from the network architecture as described in ITU-T G.8275.1, with the addition of frequency support (e.g., syntonized T-BC) being considered to improve time/phase recovery accuracy [16, 17]. The timing chain normally would be 11 hops (e.g., 10 T-BCs) and can extend to 15 hops (e.g., 14 T-BCs); this requires tight time error components control [18]. T-BC corrects the time/phase in the various network nodes and also provides a set of performance metrics including mean path delay and current offset from master [14]. As the PRTC cooperate with PRC (shown in Figure 9), the coherence between the frequency and time/phase planes can be realized, and this allows extending the time/phase holdover period during GNSS failures. Furthermore, a unified IEEE1588v2 management approach offers a compelling set of operational advantages including the ability to perform end-to-end performance analysis and troubleshooting.

## 5. Conclusion

The In-band fiber link delay asymmetry due to the use of different wavelengths in the two directions should be taken into account, especially for long haul transmission. This introduces significant time error (i.e., phase offset) within 0.3397 ns/km in* C*-band or 0.3943 ns/km in* L*-band depending on the wavelength spacing.

With the proposed scheme in this paper, the fiber link delay asymmetry can be compensated relying on the estimated mean fiber link delay by the T-BC mode and the NMS to compute the delay asymmetry in the timing chain. To deploy IEEE1588v2, bidirectional protection switch can minimize link length asymmetry in contrast to unidirectional protection switch.

It is an essential prerequisite to shorten the number of a T-BC chain, which can limit the impact of asymmetries. Furthermore, the integrity of transferring accurate time/phase synchronization over OTN and PTN with Sync-E networks can simplify network architecture, reduce OPEX, and make the network easy to maintain.

## Figures and Tables

**Table 1:** Maximum fiber link delay asymmetry for the two extreme wavelengths.

Link delay asymmetry	*C*-band 1530 nm ≤ *λ* ≤ 1569 nm	*L*-band 1570 nm ≤ *λ* ≤ 1610 nm
$\frac{A(\lambda )}{L}$	≤0.6795 ns/km	≤0.78854 ns/km
